# Phosphorylation of AKT: a Mutational Analysis

**DOI:** 10.18632/oncotarget.293

**Published:** 2011-06-10

**Authors:** Jonathan R. Hart, Peter K. Vogt

**Affiliations:** ^1^ The Scripps Research Institute, Molecular and Experimental Medicine, 10550 North Torrey Pines Road, La Jolla, CA 92037, USA

**Keywords:** Oncogenic transformation, signaling, myristylation, phosphomimetic

## Abstract

Akt (cellular homolog of murine thymoma virus akt8 oncogene) is an essential component of the PI3K (phosphatidylinositol 3-kinase) pathway. Its activity is stimulated by receptor tyrosine kinases and G-protein coupled receptors and plays a critical role in the regulation of cell proliferation, differentiation and apoptosis. A gain of function in Akt can lead to uncontrolled cell proliferation and resistance to apoptosis, both hallmarks of oncogenic transformation. In this communication, we have investigated the phosphorylation at the Akt residues T308, S473 and T450 and their roles in oncogenic transformation and signaling. We find that T450 phosphorylation has only a minimal part in these activities. In contrast, the phosphorylation of T308 and of S473 fulfills essential, distinct, and non-overlapping functions that we define with inactivating and with phosphomimetic mutations of these sites.

## INTRODUCTION

AKT (cellular homolog of murine thymoma virus *akt8* oncogene, synonym: PKB) [1-4] is a serine/threonine kinase of the AGC kinase family (PKA, PKG and PKC-related kinases) [5]; it plays a critical role in cell growth [6], differentiation [7] and survival [8]. Major targets of AKT include the TSC1-TSC2 (tuberous sclerosis) complex which mediates TORC1 (target of rapamycin complex 1) activation [6], the forkhead transcription factors FOXO1 (Forkhead box) and FOXO3a which regulate cell death, morphology and function as tumor suppressors [9-13], MDM2 (murine double minute) which regulates p53 [14] and GSK3β (glycogen synthase kinase 3β) which modulates the metabolism of the cell [15].

AKT is tightly regulated, because it controls a broad spectrum of pro-growth and pro-survival activities [16, 17]. PIP_3_, the product of PI3K (phosphatidylinositol 3-kinase), is an essential mediator of the activation of AKT by PDK1 (3-phosphoinositide-dependent protein kinase-1) [17]. The PH (pleckstrin homology) domains of both PDK1 and AKT can bind to PIP_3_, and this co-localization allows PDK1 to phosphorylate AKT at the catalytic phosphorylation site, T308 [17, 18]. AKT is also phosphorylated at the hydrophobic motif, S473, by TORC2 (target of rapamycin complex 2) and DNA-PK (DNA-activated protein kinase) [11, 17, 19, 20] and possibly by rapamycin-insensitive TORC1 [21]. S473 phosphorylation further increases enzymatic activity [22] and broadens substrate scope to include FOXO transcription factors and PRAS40 (proline-rich Akt substrate) which are not phosphorylated in the absence of S473 phosphorylation [11]. A third phosphorylation site on AKT has been identified at T450 [22]. This site is referred to as the turn phosphorylation site. It was previously thought to be a result of autophosphorylation, but more recent work has shown that its phosphorylation is controlled by TORC2 activity [23, 24]. This phosphorylation site is important in other AGC kinases, but the role in AKT signaling had not been determined.

AKT is also under the control of negative feedback loops. Activation of AKT leads to the activation of TORC1 and its downstream target p70S6K (p70 S6 kinase). p70S6K phosphorylates IRS1 (insulin receptor substrate). This event lowers PI3K activity, attenuating the phosphorylation of AKT at T308 [25]. Another mechanism for controlling AKT depends on the modulation of TORC2 activity. TORC2 activity requires the intact and unphosphorylated TSC1-TSC2 complex which is disrupted by AKT phosphorylation [26]. This inhibition of TSC1-TSC2 leads to a decrease in S473 phosphorylation. TORC1 and TORC2 activities are also suppressed by DEPTOR. Suppression of both TOR complexes leads to situations where DEPTOR can act either as a suppressor or activator of AKT [27]. These mechanisms assure stable self-attenuating signaling by Akt.

AKT is commonly activated in human cancers where it stimulates cell proliferation and survival [28, 29]. Aberrant, cancer-specific activation of AKT can be caused by several distinct mechanisms. Mutated or overexpressed receptor tyrosine kinases can stimulate the entire PI3K pathway. Mutations in both catalytic (p110) or regulatory (p85) subunits of PI3K or in PTEN (phosphatase and tensin homolog) can also generate elevated levels of PIP_3_, leading to constitutive activation of AKT. In some cases, AKT itself is constitutively active because of a mutation in the PH domain (E17K) [30] or, as in the case of the v-AKT of murine thymoma virus, fusion to viral Gag sequences that mediate membrane attachment [1]. The latent oncogenicity of AKT can also be revealed by fusing an amino terminal myristylation sequence to the protein, thus inducing constitutive, PIP_3_-independent membrane localization [31]. Alteration of the specificity of the PH domain towards the abundant PI(4,5)P_2_ is sufficient to lead to constitutive activation and oncogenicity of Akt suggesting that other mutations of the PH domain may occur [32].

We have systematically mutated the phosphorylation sites in AKT, T308, T450 and S473, to either alanine (A) which cannot be phosphorylated, or to aspartate (D) which also cannot be phosphorylated, but mimics phosphorylated serine. We have then determined the effect of the mutations on the oncogenic and signaling potentials of the protein.

## MATERIALS AND METHODS

### Plasmids

pBSfi-Akt and pBSfi-myr-Akt of mouse *Akt1* have been previously described and contain a C-terminal HA tag [31]. These were mutated by site directed mutagenesis at T308, T450 and S473 with the following primers and their complement:T308D, “TGCCACTATGAAGGATTTCTGCGGAACGCCG”, T308A, “TGCCACTATGAAGGCATTCTGCGGAACGCCG”, S473D, “CACTTCCCCCAGTTCGACTACTCAGCCAGT GGC”, S473A, “CACTTCCCCCAGTTCGCCTACTCAGC CAGTGGC”, T450D, “CAGCTCAGATGATCACCATCGACCCGCCTGATCAAGATGACAG”, T450A, “CAGATGATCACCATCGCGCCGCCTGA TCAAG”, MYR-G2A, “TATCGATAAGCTTATGGCGAGCAGCAAGAGCAAGC”, R25A, “CTTGAGGAGGAAGTAGGCTGGCCGCCAGGTTTT”. The resulting plasmids were subcloned into the SfiI site of RCAS(A).sfi. In the experiments described in this manuscript, AKT refers to the protein encoded by the mouse *Akt1* gene.

### Assays for oncogenic transformation

RCAS(A) constructs were transfected into chicken embryonic fibroblasts (CEF) using the Lipofectamine 2000. 24 hours after transfection, the growth medium was removed and replaced with a nutrient agar overlay. Additional nutrient agar was added every 3 days for 2 weeks. The agar was removed and the cells stained using crystal violet.

### Western Blotting

RCAS(A) constructs were transfected into CEF using the polybrene/DMSO shock method [33]. CEF were cultured in cloning media for 3 passages during which time the virus infected all the cells as monitored by RCAS(A)-GFP control. Cells were lysed using CHAPS lysis buffer (50 mM TrisHCl (pH 7.5), 1 mM EDTA, 0.3% (w/v) CHAPS, 1 mM sodium orthovanadate, 10 mM sodium β-glycerophosphate, 50 mM sodium fluoride, 5 mM sodium pyrophosphate, 0.15 M NaCl, 0.1 mM PMSF and complete protease-inhibitor cocktail (1 tablet/50mL lysis buffer)). Protein lysates were collected, left on ice for 30 minutes and centrifuged at 16,000xg. Protein concentrations were measured using the BCA method. The protein concentrations were normalized to 15 μg per sample and separated on a 4-12% Bis-Tris Nupage Gel (Invitrogen, Carlsbad, CA) using a MOPS running buffer. Proteins were transferred to Millipore Immobilon P PVDF membranes. Membranes were blocked with 5% BSA in TBS-T and probed using antibodies against p-T308-Akt (#9275), p-T450-Akt (#9267), p-S473-Akt (#9271), p-S6 (#2211), p-GSK3β (#9336), HA (#2367), actin (#4976), FOXO1 (#9462) (Cell Signaling, Danvers, MA). Membranes were rinsed three times with TBS-T and probed with goat anti-rabbit-HRP or goat anti-mouse-HRP as appropriate. The membranes were rinsed an additional three times and developed using SuperSignal West Pico ECL substrate and recorded using a Biorad Chemidoc XRS camera.

## RESULTS

### Mutating AKT phosphorylation sites

The aim of this mutant analysis was to obtain comprehensive data on the significance of individual AKT phosphorylation sites for oncogenicity and for signaling. The mutations were introduced both into wild-type AKT and into the constitutively active, myristylated AKT to identify activating as well as inactivating changes and to delineate the role of membrane localization in the functions of Akt. Wild-type and mutated AKT proteins were expressed as inserts in the avian retroviral vector RCAS [34], and all constructs included a C-terminal HA epitope tag for detection. Comparison between tagged and untagged versions of the same protein did not detect any tag-related functional differences. RCAS constructs were transfected into chicken embryo fibroblasts (CEF) using Lipofectamine 2000 ® (Invitrogen, Carlsbad, CA). Oncogenic activity was determined by enumerating foci of transformed cells on CEF monolayers. For the analysis of AKT-mediated signaling, cultures in which each cell expressed the transfected construct were harvested, and cell lysates from these harvests were used for Western blot analyses to verify the phosphorylation status of AKT and to determine signaling activity. For this latter purpose, GSK3β was probed as a direct AKT target, and phosphorylation of the ribosomal protein S6 was used as read-out for downstream signaling activity that proceeds through the TSC complex, RHEB (Ras homolog enriched in brain) and TORC1 to p70S6K.

### The catalytic phosphorylation site, T308

**Figure 1:**
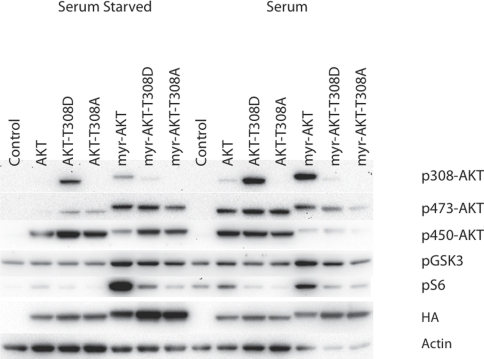
Western blots of T308 catalytic phosphorylation site mutants The T308 phosphorylation site was mutated to alanine (T308A) or aspartate (T308D) in both myristylated and wild-type constructs. Akt phosphorylation and the downstream targets, GSK3β and S6 ribosomal protein are shown. Actin was used as a loading control, and HA is used to detect the overexpressed constructs. The T308A mutation blocks downstream signaling. The T308D mutation generates an incomplete mimic of phosphoserine and results in decreased signaling of myr-Akt-T308D.

Phosphorylation of T308 is essential for catalytic activity of Akt. The active site of Akt is disordered in the absence of phosphorylation [35]. Phosphorylation of T308 allows the organization of a network of interactions including stabilization of the αC helix that is essential for the functioning of the catalytic domain and formation of the active site [35]. A mutation of this site is therefore expected to have a negative effect on AKT functions, and this is confirmed in the transformation and signaling studies (Table 1, Fig. 1). The non-myristylated T308D mutation shows an extremely low transforming activity, and the non-myristylated T308A is inactive in transformation assays. In the myristylated version, T308D significantly reduces transforming potential, and T308A completely abolishes it. This loss of activity is also reflected in reduced signaling. The non-myristylated T308 mutants do not change phosphorylation of GSK3β from the levels seen in wild-type AKT or control cells that have received empty vector, either in the presence or absence of serum. Phosphorylation of S6 is suppressed compared to wild-type AKT and is close to the levels seen with empty vector. In the myristylated versions of these mutations, T308D shows great loss in signaling to S6 in the presence or absence of serum and a minor reduction in GSK3β signaling that becomes evident in the presence of serum. The myristylated T308A mutant shows an even greater reduction of S6 signaling independent of serum. The enhanced phosphorylation of GSK3β by this mutant under conditions of serum starvation is surprising and puzzling, but may derive from an indirect effect of the mutant on the activation of endogenous Akt. S473 phosphorylation is slightly increased, however the catalytically important T308 phosphorylation is still low. This apparent enhancement of Akt signaling is lost upon serum stimulation and will therefore not affect transformation which takes place in the presence of 3% serum. The low oncogenic and signaling activity of the T308D mutants is largely a reflection of the incomplete mimicking of phospho-threonine by aspartate. These results support the conclusion that phosphorylation of T308 in AKT is essential and necessary for the cell-transforming activity and for signaling.

**Table 1:** Efficiencies of oncogenic transformation The table lists the number of foci induced, standardized to 1 ng DNA. The standard error for these focus determinations is 6 %. All constructs are expressed in CEF with the RCAS vector.

Construct	Foci/ng DNA
Vector only	0
AKT	0
myr-AKT	1700
AKT-T308D	5
AKT-T308A	0
myr-AKT-T308D	450
myr-AKT-T308A	0
AKT-S473D	500
AKT-S473A	0
myr-AKT-S473D	2400
myr-AKT-S473A	2000
Akt-R25A	0
Akt-R25A,S473D	28
AKT-T450D	12
AKT-T450A	20
myr-AKT-T450D	1700
myr-AKT-T450A	1300

### The hydrophobic phosphorylation site, S473

**Figure 2:**
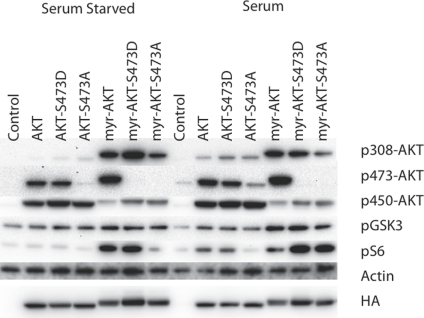
Western blots of S473 hydrophobic motif phosphorylation site mutants The T450 phosphorylation site was mutated to alanine (S473A) or aspartate (S473D) in both myristylated and wild-type constructs. Akt phosphorylation and the downstream targets, GSK3β and S6 ribosomal protein are shown. Actin was used as a loading control, and HA is used to detect the overexpressed constructs. The S473D mutation significantly enhances transformation by both the wild-type and myristylated constructs.

The cell-transforming activities of the S473D and S473A mutations are shown in Table 1. Whereas the S473A mutant in the non-myristylated protein is inactive, the S473D mutant shows highly significant transforming activity. In the myristylated version, these mutations do not strongly affect the base levels of activity seen with the myristylated wild-type AKT. Both myristylated S473 mutants remain strongly transforming. The signaling activity of the mutants is not fully in accord with the transforming activity (Fig. 2). The non-myristylated S473D, despite its transforming activity, shows only slightly elevated phosphorylation of AKT T308 in the presence, but not in the absence of serum, and does not significantly stimulate the phosphorylation of downstream targets GSK3β and S6 independent of growth factor availability. Under serum-starved conditions, the myristylated S473D mutant shows enhancement of phosphorylation on AKT T308 and S6, but not on GSK3β. In the presence of serum, there is no effect on AKT T308, but phosphorylation of S6 is further increased. The myristylated S473A mutant induces phosphorylation of AKT T308 in the presence or absence of serum. In the presence of serum, in agreement with its transforming activity, the myristylated S473A also strongly signals to S6. The observations on the non-myristylated S473D mutant show that phosphorylation at this site can activate a latent oncogenicity. However, the persistent activities of the myristylated S473A mutant show that this phosphorylation is not essential for signaling through TORC1 or for oncogenicity that is activated by constitutive localization to a cellular membrane.

The transformation by S473D raises the possibility of Akt activation by a mechanism analogous to the activation of other AGC (cAMP-dependent protein kinase/protein kinase G/protein kinase C) kinases such as p70S6K or SGK [36, 37]. In these kinases, phosphorylation of the hydrophobic motif mediates the binding to PDK1, allowing phosphorylation of the catalytic site. This association is independent of PH domain binding. We therefore combined the PH domain-inactivating mutation R25A with S473D [31]. The combination of R25A with S473D strongly reduces the transforming ability of Akt, although it does not eliminate it. The residual transforming activity may reflect the direct interaction of PDK1 with the S473D mutant. However, the strong reduction of transforming potential indicates that the S473D mutation is still dependent upon membrane binding for activity.

### The turn phosphorylation site, T450

**Figure 3:**
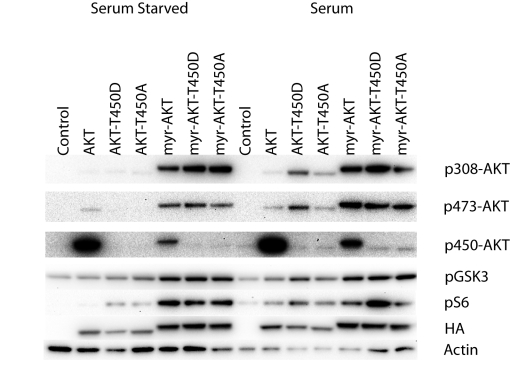
Western blots of T450 turn motif phosphorylation site mutants The T450 phosphorylation site was mutated to alanine (T450A) or aspartate (T450D) in both myristylated and wild-type constructs. Akt phosphorylation and the downstream targets, GSK3β and S6 ribosomal protein are shown. Actin was used as a loading control, and HA is used to detect the overexpressed constructs. Mutation of T450 did not block downstream signaling but may slightly enhance signaling induced by serum stimulation.

T450 was mutated to A or D in both wild-type and myristylated AKT. The effect of the mutations of the wild-type and myristylated proteins is minimal on signaling, shown in Figure 3, or transformation, summarized in Table 1. A slight change in signaling is seen in the presence of serum and growth factors. The T450D mutation in wild-type and in myristylated AKT leads to an enhanced phosphorylation of the S6 ribosomal protein, presumably mediated by an upregulation of signaling through TORC1. The elevated signaling activity characteristic of myristylated AKT is not affected by mutation of the T450 site. The T450A and T450D mutations also fail to inactivate the strong cell-transforming activity of myristylated AKT. Surprisingly, in the wild-type protein, these mutations induce a marginal, but clearly detectable transforming activity (Table 1). There is also slightly increased phosphorylation of GSK3β by the T450D mutation in the wild-type protein. Studies on other AGC kinases have implicated the turn phosphorylation site in the control of kinase activity, substrate scope and protein stability [23, 24]. However, the mutations of T450 do not substantially change cell transforming and signaling activities of Akt and do not have a strong effect on steady-state protein levels. We conclude that the turn phosphorylation site does not play a significant role in the control of Akt signaling and oncogenicity.

## DISCUSSION

In the standard progression of events leading to AKT activation, the PH domain binds to PIP_3_, facilitating association with PDK1. This leads to phosphorylation on T308 and an initial activation of catalytic activity. Separately, TORC2 is activated and phosphorylates AKT at S473, inducing an additional gain in the catalytic activity of AKT. Alternatively, TORC2, activated in response to growth factor signaling, phosphorylates AKT at S473 first, followed by the binding of phosphorylated hydrophobic motif to the PIF (protein kinase C-related kinase 2-interacting fragment)-binding pocket of PDK1. Upon binding of Akt to PDK1, phosphorylation of AKT on T308 ensues. The two mechanisms are not mutually exclusive; the prevalence of one or the other may be context-dependent. Both mechanisms employ three basic components, membrane binding, PDK1 phosphorylation of T308 and TORC2 phosphorylation of S473.

Membrane localization of AKT can occur in several ways. In response PIP_3_ produced by PI3K, AKT localizes to the membrane. Additionally, modification of AKT by myristylation, Gag-fusion or mutation of the PH domain can drive AKT to the membrane constitutively. All of these mechanisms result in phosphorylation and activation of AKT at both T308 and S473. PDK1 has a high affinity to membrane lipids, specifically those containing PIP_3_ but also PI(3,4)P_2_ and PI(4,5)P_2_. Consequently, even under quiescent conditions, a substantial fraction of PDK1 remains membrane associated. If AKT is brought to the membrane, whether by myristylation, Gag fusion or PH domain mutation, AKT will be activated by virtue of this co-localization. It has been suggested that the PH domain serves as a mask of the T308 phosphorylation site [38, 39]. This arrangement would prevent the accidental activation of AKT in the event of a chance collision between PDK1 and AKT. Interaction of the PH domain with lipid would open access for PDK1 to T308, and this conformational change appears to be a prerequisite to AKT activation. However, when myristylation mediates PH domain-independent membrane localization, the frequency of encounters with PDK1 could be high enough to drive phosphorylation of T308. It is also possible that the attachment of a peptide to the N-terminus destabilizes the interaction of the PH domain with the catalytic domain. As yet there is no structure of the Akt protein showing the interaction of the PH domain and catalytic domain in the inactive protein. Mutation of the critical glycine of the myristylation tag to an alanine completely blocks transformation, suggesting that N-terminal additions do not inherently destabilize the inhibitory PH-domain conformation.

**Figure 4:**
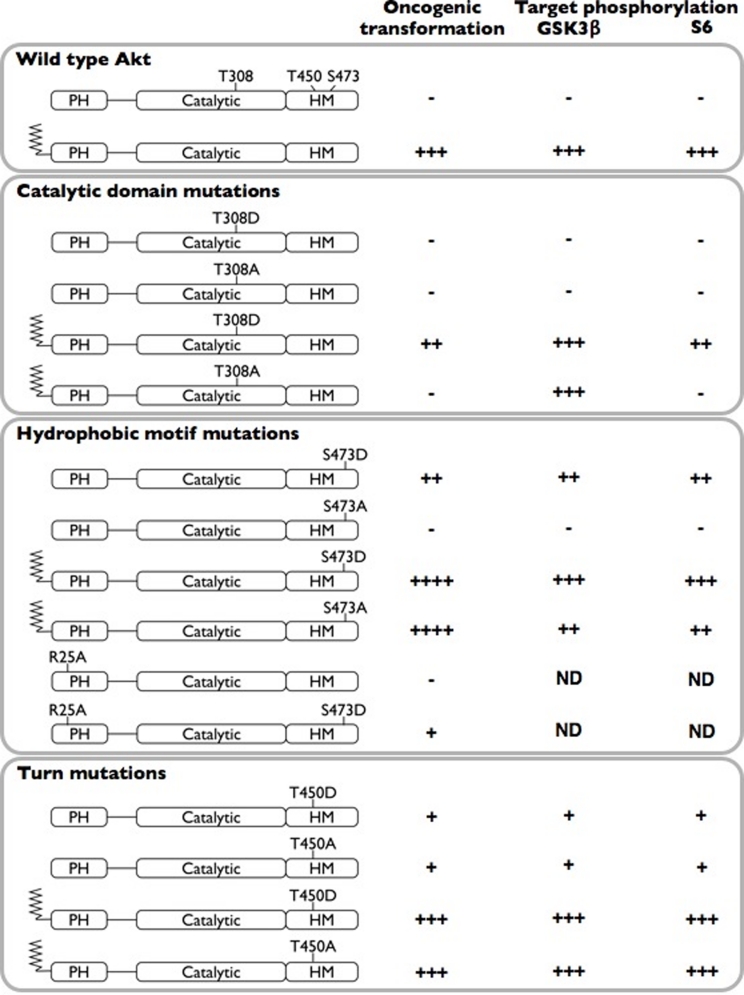
Summary of phosphorylation site mutations: effects on Akt-induced oncogenic transformation and signaling Oncogenic transformation was arbitrarily graded as follows: “-” (<10 foci/ng DNA), “+” (10 to 50 foci/ng DNA), “++” (50 to 500 foci/ng DNA), “+++” (500 to 1999 foci/ng DNA) and “++++” (2000 and more foci/ng DNA). The density of the bands in the Western blots was similarly assessed as “-” (no band), “+” (weak target phosphorylation), “++” (intermediate target phosphorylation), and “+++” (strong target phosphorylation). PH, pleckstrin homology domain; HM, hydrophobic motif.

Mutation of AKT phosphorylation sites results in very different effects on oncogenicity and on signaling (Fig. 4). Not unexpectedly, the T308 site emerges as the most critical for these activities. We find that the T308D is an unsuitable substitution for the native phospho-threonine. AKT can exist in multiple conformational states, catalytically active and inactive. AKT that is not phosphorylated at T308 is predominantly in the inactive state. Phosphorylation of T308 results in the formation of an ordered hydrogen bonding network between the catalytic N lobe and the activation loop. This hydrogen bonding network between the phosphate of the phospho-threonine, histidine 195 and arginine 274 orders the active site of the enzyme, allowing for both ATP and substrate to bind. The substitution of aspartate for threonine does not bring the appropriate number of hydrogen bond acceptors or the correct conformation (carboxylic acids being trigonal planar vs phosphates being tetrahedral) into this critical organizing structure. The imperfect mimic of the phosphorylated state results in a partial activation of the protein, but the activity is less than the native structure. Thus, the substitution causes a loss of transformation and signaling activity in myr-T308D as compared to the parent myristylated protein. The transforming potential of the non-myristylated T308D mutant is barely detectable.

This imperfect mimicry also leads to decreased activity of myr-Akt-T308D-S473D as compared with myr-Akt and myr-Akt-S473D and decreased activity of Akt-T308D-S473D as compared with Akt-S473D (data not shown). The use of phospho-mimetic aspartate at these sites should be discouraged as they do not recapitulate a fully active form of Akt.

Phosphorylation of S473 is required for maximal activation of AKT. In the wild-type AKT protein, this phosphorylation leads to further stabilization of the N-lobe of the kinase. The entire regulatory domain wraps around the AKT kinase with the phosphorylated S473 nestled atop the α helix. This stabilization pushes the equilibrium toward the active conformation, increasing AKT catalytic activity by approximately 10 fold [22]. The interaction is simulated quite well by the S473D mutation. This mutation alone is sufficient to render wild type AKT oncogenic. The S473D mutant of AKT has been previously shown to be a better substrate for PDK1 through direct physical interaction with the PIF-binding pocket on PDK1 [40]. This mechanism is central in the activation of other AGC kinases, but its need is bypassed in AKT by PIP_3_ mediated membrane interaction. However, we find that the PH domain still plays a significant role in the activation of S473D as the introduction of the R25A mutation blocks most of the transforming activity of S473D. These results support the hypothesis that the PH domain is not only responsible for localization to the membrane but also controls the accessibility of the T308 site [38, 41].

The limiting factor in downstream signaling from serum-stimulated or myristylated Akt is probably the phosphorylation of S473. The level of S473 phosphorylation is controlled by TORC2 and PHLPP. The phosphomimetic S473D mutation eliminates this control and thus permits enhanced signaling to S6.

Despite this well documented role of S473 phosphorylation, we find that it may not be necessary for transformation. In the myristylated construct, both S473D and S473A mutations are active and transform with comparable efficiency. Myristylated AKT is highly phosphorylated at both T308 and S473, and S473D does not further increase the transformation induced by the myristylated protein. However, the strong transforming activity of the myristylated S473A mutant suggests that S473 phosphorylation is not essential for the oncogenic activity of AKT. S473 has been previously shown to control two separate aspects of AKT. First, S473 phosphorylation causes the hydrophobic motif to bind to the αC helix in the catalytic domain of AKT and to further stabilize the active form of the kinase [35]. Second, S473 results in a broadening of the substrate scope of AKT to include more diverse targets such as the forkhead transcription factors and PRAS40 [11]. We find that under the conditions of our assays, FOXO1 and FOXO3a are destabilized by myr-AKT-S473A (Supplemental Figure 1). This is in contrast to previous experiments which used knockout of TORC2 components to limit S473 phosphorylation [11]. However, in our experiments AKT is overexpressed, and the higher levels of the enzyme may be adequate to phosphorylate FOXO despite reduced catalytic activity.

In the T308D and myr-T308D-expressing cells, the protein detected by the phospho-T308 Akt antibody is likely the endogenous wild-type protein. This interpretation is suggested by the observation that both T308D and myr-T308D have similar expression by HA blotting, but have drastically different phospho-T308 reactivity. The HA blotting also documents the expected mobility shift from the myristylation; that shift is absent from the phospho-T308 blot. This suggests that T308D constructs protect endogenous Akt from dephosphorylation by PP2A rather than directly participating in downstream signaling.

Our experiments with the T450 mutation suggest that this site plays a minimal role in the transforming and signaling activities of AKT. The T450 phosphorylation site is disordered in all the structures of AKT obtained so far, but a model of the potential structure has been generated [42]. Knockout of TORC2 components causes a loss of phosphorylation of T450, but it is not clear whether TORC2 phosphorylates T450 directly or controls the activity of another kinase [43]. These experiments also showed that in the absence of TORC2, AKT is unstable and readily degraded. We did not note a significant elevation or reduction of AKT levels with the T450D or T450A mutants in our experiments. It is possible that overexpression masks a rapid turnover of the protein. T450 has also been reported to be a substrate of JNK (c-jun N-terminal kinase) [44]. In those experiments it was found that T450 phosphorylation can lead to increased T308 and S473 phosphorylation as well as AKT activity and stability. Our results show that there is a slight activation of oncogenic activity, but it is much less than in other strongly transforming constructs.

There are significant differences in the expression levels of several Akt constructs. Specifically myr-Akt-T308D and myr-Akt-T308A are expressed at a much higher level than myr-Akt without mutation. These changes in protein expression can be attributed to previously identified changes in Akt protein stability [45-47]. In a previous publication, we have also described enhanced proteolytic cleavage of membrane bound and strongly transforming forms of Akt [32]. These observations suggest the existence of a negative feedback loop that is triggered by hyperactive Akt and is capable of regulating Akt at the level of protein expression.

## SUPPLEMENTAL FIGURES


