# Supplementary figures and images for: Phosphorylation of AKT: a Mutational Analysis
Source: Oncotarget. 2011 Jun 10;2(6):467–76. doi: 10.18632/oncotarget.293 (PMC3139455; doi:10.18632/oncotarget.293)

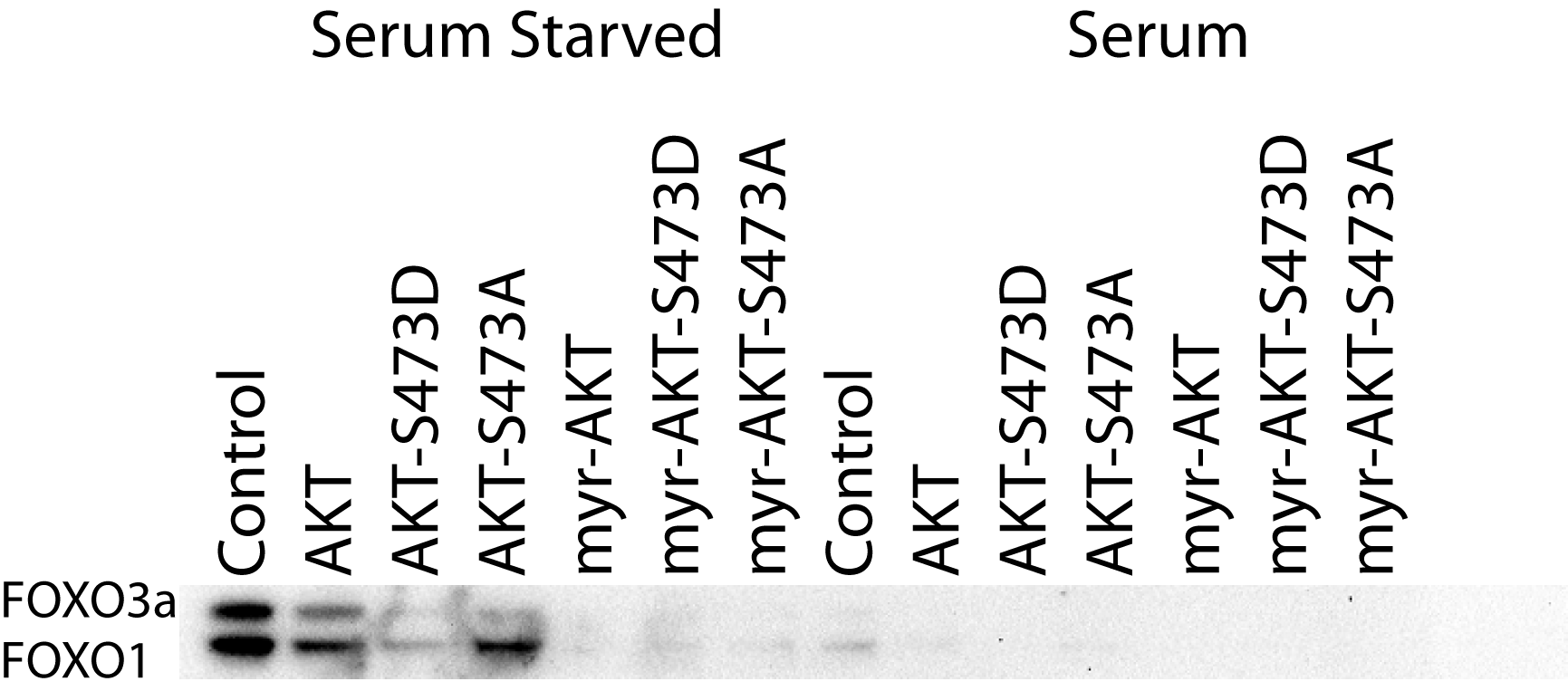

Supplement: Supplementary file 1 [file oncotarget-02-467-s001.tif]
